# Supplementary material for: Socioeconomic disparities in diabetes prevalence and management among the adult population in Bangladesh
Source: PLoS One. 2022 Dec 20;17(12):e0279228. doi: 10.1371/journal.pone.0279228 (PMC9767371; doi:10.1371/journal.pone.0279228)
Supplement: S1 File — (DOCX) [file pone.0279228.s001.docx]

**Supplementary Materials**

**Fig S1. Flowchart of analytic sample, BDHS 2017-18**

**
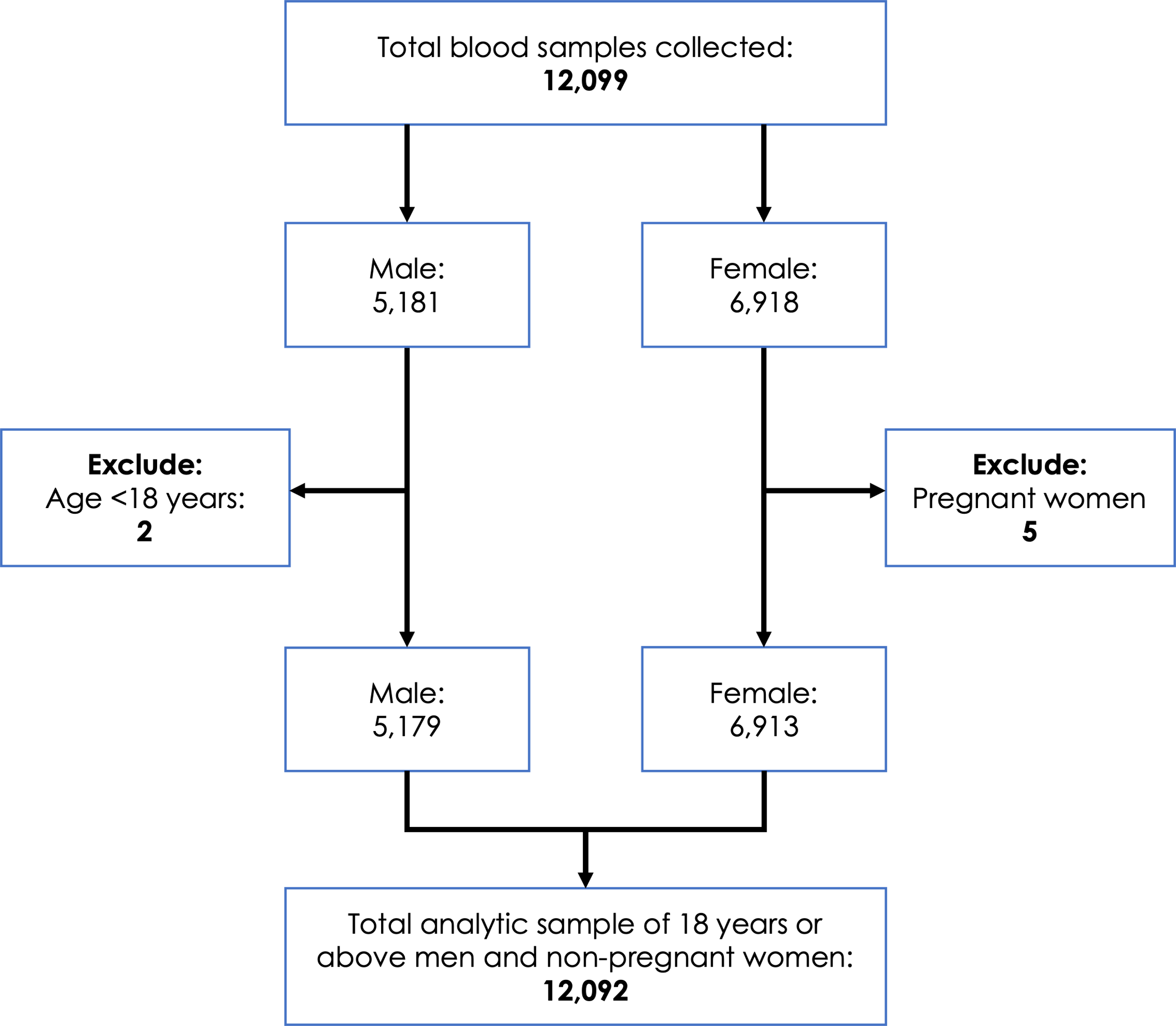
**

**Table S1: Characteristics of sample and response cases, BDHS 2017-18**

| **Background characteristics** | **Precedent distribution of sampled and response-cases by characteristics (unweighted)** | | | |
| --- | --- | --- | --- | --- |
|  | *Sample* | *Response* | *Difference* | *p-value* |
| **Age** |  |  |  |  |
| 18-34 | 44.9 | 44.5 | 0.3 | 0.510 |
| 35-44 | 19.7 | 20.0 | -0.3 | 0.538 |
| 45-54 | 14.0 | 14.0 | 0.1 | 1.000 |
| 55-64 | 11.3 | 11.3 | 0.0 | 1.000 |
| 65+ | 10.1 | 10.1 | 0.0 | 1.000 |
|  |  |  |  |  |
| **Sex** |  |  |  |  |
| Male | 45.5 | 43.1 | 2.4 | <0.001 |
| Female | 54.5 | 56.9 | -2.4 | <0.001 |
|  |  |  |  |  |
| **Education** |  |  |  |  |
| No education | 24.5 | 24.8 | -0.2 | 0.569 |
| Primary | 29.5 | 30.1 | -0.6 | 0.283 |
| Secondary incomplete | 24.8 | 25.2 | -0.4 | 0450 |
| Secondary complete or higher | 21.2 | 19.9 | 1.3 | 0.009 |
|  |  |  |  |  |
| **Wealth index** |  |  |  |  |
| Quintiles I and II | 37.0 | 38.4 | -1.4 | 0.018 |
| Quintile III | 19.0 | 19.8 | -0.7 | 0.098 |
| Quintiles IV and V | 44.0 | 41.8 | 2.2 | <0.001 |
|  |  |  |  |  |
| **Locality** |  |  |  |  |
| Urban | 37.5 | 35.7 | 1.8 | 0.002 |
| Rural | 62.5 | 64.3 | -1.7 | 0.002 |
|  |  |  |  |  |
| **Total** | **100.0** | **100.0** | **-** | **-** |
|  |  |  |  |  |
| **Number of respondents** | **14,704** | **12,301** | **-** | **-** |
|  |  |  |  |  |
| **Non-response rate (%)** | **-** | **16.3** | **-** | **-** |
