# Supplementary material for: Socioeconomic disparities in diabetes prevalence and management among the adult population in Bangladesh
Source: PLoS One. 2022 Dec 20;17(12):e0279228. doi: 10.1371/journal.pone.0279228 (PMC9767371; doi:10.1371/journal.pone.0279228)
Supplement: Attachment — Submitted filename: Response to reviewers_R1.docx [file pone.0279228.s002.docx]

22 August 2021

Mohammad Bellal Hossain, PhD
Academic Editor

PLOS ONE

San Francisco, CA 94111, United States

Subject: Revision of the manuscript PONE-D-21-02392

Dear Dr. Hossain:

We would like to submit the revised version of our research article titled “Socioeconomic disparities in diabetes prevalence and management among the adult population in Bangladesh”. The revised version addresses all the comments by the editor and two reviewers. We have made necessary changes as instructed in the paper in track changes. We would also like to take the opportunity to thank the reviewers for their insightful comments.

All the authors have approved this revised version of the manuscript and contributed towards the revision. The authors do not have any conflicts of interest to disclose.

Please find the reviewers comments and our responses below:

**Comments from the Editor**

1. The manuscript has lost its focus; it has failed to comply with the title. It its current format, the manuscript looks like a monograph though PLOS ONE has not problem with space. I do not see any justification for keeping the following sections in the manuscript: Correlates of diabetes among women of reproductive age; and Changes in correlates of diabetes between 2011 and 2017-18 among older adults. Please consider removing these two sections to make a focused manuscript.

**Authors’ Response:** The sections “Correlates of diabetes among women of reproductive age” and “Changes in correlates of diabetes between 2011 and 2017-18 among older adults” are dropped (p.18, line 342 to p.20, line 392 in the revised manuscript with track changes). Abstract, Methods, and Discussion sections have been updated accordingly.

2. The tables should provide the ‘n’ in any format to demonstrate the sample size f different outcome variables.

**Authors’ Response:** Updated Table 3 (p.16) by adding ‘n.’

3. Please ensure that your manuscript meets PLOS ONE’s style requirements, including those for file naming. The PLOS ONE style templates can be found at

<https://journals.plos.org/plosone/s/file?id=wjVg/PLOSOne_formatting_sample_main_body.pdf> and

<https://journals.plos.org/plosone/s/file?id=ba62/PLOSOne_formatting_sample_title_authors_affiliations.pdf>

**Authors’ Response:** The manuscript has been reviewed to ensure that it meets PLOS ONE style requirements.

4. Please provide additional details regarding participant consent. In the ethics statement in the Methods and online submission information, please ensure that you have specified what type you obtained (for instance, written or verbal, and if verbal, how it was documented and witnessed). If your study included minors, state whether you obtained consent from parents or guardians. If the need for consent was waived by the ethics committee, please include this information.

**Authors’ Response:** We added a subsection on ethics approval under the Methods section (p.11, lines 233–239).

**Comments from Reviewer 1**

1. As suggested by the IDF, we cannot label respondents as diabetic patients. It should be, ‘people with diabetes’. Please change throughout the manuscript.

**Authors’ Response:** We thank the reviewer for highlighting this important point. We have replaced ‘diabetics/diabetic patients’ with ‘people with diabetes’ throughout the manuscript.

2. The current title is not consistent with the way the authors analyse and reported data. According to the title, the authors should consider socioeconomic status (SES) as exposure and adjust all the confounders rather than putting all of them in kitchen sink regression model.

**Authors’ Response:** As per the manuscript’s title, we consider SES as exposure and have built the multivariate model in a stepwise approach (viz., first running the bivariate tables, then putting the potential confounders one by one, testing interactions between SES and the cofounders, and using the goodness of fit test to decide on the final list of variables). We believe that the statistical measures and approach used in the analysis are appropriate to achieve the objectives of the study. In addition, the title also aligns with the analysis, results found, and possible explanations of the findings.

3. The manuscript is written poorly with a lack of clear direction. For example, the focus of the manuscript is on the socioeconomic disparities in diabetes prevalence, however, most of the literature are not in relation to the issue. Secondly, the authors defined type2, type1, GDM and provided every detail of global prevalence which are not needed, while diabetes management, an important part of the manuscript, is completely ignored.

**Authors’ Response:** We are surprised that the reviewer feels this way. Out of 6 paragraphs in the introduction section, only one paragraph broadly discussed the global context before getting into the country context. Also, diabetes management and its relation to socioeconomic disparity are noted based on the literature review (p.6, lines 123–128), so it was not completely ignored.

4. It is not appropriate to investigate the changes of risk factors, especially for time variants factors e.g. age, BMI, and hypertension status, when they modelled each survey data separately.

**Authors’ Response:** Following the Editor’s recommendation, we dropped the section on “Changes in correlates of diabetes between 2011 and 2017-18 among older adults.”

5. The BMI should be categorised based on Asian cutoff as suggested by the WHO expert consultation due to the high risk of type 2 diabetes and cardiovascular disease in Asian people at lower BMIs than the existing WHO cutoff. However, it is not clear whether the authors used Asian or WHO cut-off. About ~17 % of the study population is underweight that has differential impact on diabetes. So, I suggest not to combine underweight and normal weight.

**Authors’ Response:** We agree that some literature has used different BMI categories, particularly for studies in Asia. We, however, decided to use the most widely used and internationally accepted categories of BMI for our analysis. In addition, the Government of Bangladesh also uses these BMI cut-offs for program monitoring and strategic planning purposes.

We re-analyzed data without combining underweight and normal BMIs and updated the manuscript accordingly.

6. It is not appropriate to combine wealth quintiles.

**Authors’ Response:** We respectfully disagree with the reviewer’s comment. DHS asset index is a relative index for comparing households or individuals within one survey. Combining wealth quintiles or creating quantiles/terciles from the asset index to differentiate the poor and non-poor respondents is a common, standard practice and published many times in reputable journals (including PLOS ONE). Our re-categorization of the quintiles was primarily based on both old^[1]^ and new^[2]^ study findings, which showed that the households in the two lowest wealth quintiles can be considered as the ‘low socioeconomic status’ for a lower-middle-income country like Bangladesh.

7. It is unclear whether the authors checked multi-collinearity, interaction or effect modification in the data.

**Authors’ Response:** We checked for multicollinearity, interactions between SES and the covariates, and effect modifications in the dataset. We added texts describing this in pp.9–10, lines 203–208.

8. When the overall prevalence of diabetes is >10% (especially for table 5). For such a common outcome, logistic regression (odds ratio) overestimates or jeopardizes the association. So, the adjusted association (Odds ratio) might be just by chance, not the true association. Prevalence ratio is the suitable option for a cross-sectional study with a common outcome, while the odds ratio is the best option for a case-control study or the rare outcome. Authors can consult with log-binomial or other advanced methods for calculating adjusted prevalence ratio.

**Authors’ Response:** Following the Editor’s recommendation, we dropped the section on “Changes in correlates of diabetes between 2011 and 2017-18 among older adults.” The overall prevalence of diabetes in our main analysis remains under 10%, and therefore, we reported the outcomes using logistic regression models.

9. Please provide flowchart of analytic sample. The final report shows that total sample was 12099 (table 13.5.1: women 6918, table13.5.4:men 5181), including pregnant women. However, your analytic sample is 12092 which is only 7 women less compared to the national report.

**Authors’ Response:** We provided a flowchart in the supplement (Fig S1).

10. For subgroup analysis of women, number of pregnancies/parity should be adjusted in the model.

**Authors’ Response:** Following the Editor’s recommendation, we dropped the section on “Correlates of diabetes among women of reproductive age.”

11. For table 2, please report overall numerator and denominator.

**Authors’ Response:** Table 2 already has the denominator (number of respondents) reported. We tried putting the frequencies in each cell, but then the table becomes hard to read. No changes were made.

12. The discussion is sloppy. Policy suggestions for diabetes management need to be compact as currently reported as literature review.

**Authors’ Response:** We made some adjustments to streamline the discussion section. We would be benefitted from specific comments on how the discussion section can be improved instead of a sweeping statement from the reviewer.

13. The manuscript must be edited by a native speaker.

**Authors’ Response:** The manuscript has a native English-speaking and at least one co-author highly proficient in English, and they thoroughly reviewed the manuscript. We request the reviewer to point out the exact places in the revised manuscript where it is felt that further correction is needed to meet the desired standards.

**Comments from Reviewer 2**

1. General comments would be to talk about people with diabetes rather than diabetics. I understand from clinical colleagues that there is a move away from labelling people based on their disease status.

**Authors’ Response:** We thank the reviewer for highlighting this important issue. We have replaced ‘diabetics/diabetic patients’ with ‘people with diabetes’ throughout the manuscript.

2. The sample size is small for doing multiple comparisons, and this should be acknowledged.

**Authors’ Response:** Following the Editor’s recommendation, we dropped the section on “Changes in correlates of diabetes between 2011 and 2017-18 among older adults.” The analysis in the revised manuscript is based on a representative sample of the adult population in Bangladesh for 2017-18.

3. Please give exact p-values unless p<0.0001.

**Authors’ Response:** Table 3 has been revised following the PLOS One’s submission guideline. We reported exact p-values for all values greater than or equal to 0.001 and p-values less than 0.001 were expressed as p<0.001.

4. There are probably >100 p-values in this paper and so it is inappropriate to focus on any <0.05.

**Authors’ Response:** After dropping the additional analyses as per the Editor’s recommendation, the number of p-values has declined substantially. No changes were made.

5. Have the authors considered doing a global test for heterogeneity in their tables?

**Authors’ Response:** Testing for heterogeneity is important for meta-analysis or panel data analysis. It helps to deal with different study designs, different interventions, etc., and to take necessary analytical actions. However, following the Editor’s recommendation, our revised manuscript relies on one nationally repetitive cross-sectional survey data. We, therefore, respectfully disagree with the reviewer that performing a test for heterogeneity is essentially applicable to our analysis.

6. On a similar note, it would be more appropriate to calculate tests for trend for factors that show a trend (eg SES, education, etc).

**Authors’ Response:** Following the Editor’s recommendation, we dropped the section on “Changes in correlates of diabetes between 2011 and 2017-18 among older adults.” The revised manuscript no longer compares the trends over two time points.

7. Please describe ORs consistently. On line 278, the OR is described as being 31% higher and 2.4 times higher. We do not normally refer to ORs in terms of percentages. Similarly, on line 258 they are referred to as likelihood.

**Authors’ Response:** We updated the texts (in pp.15–17) in line with the reviewer’s comment.

8. In the Introduction, line 107, please talk about a decline in the probability of dying prematurely (since the probability of dying=1 in all countries).

**Authors’ Response:** We made the suggested change (p.5, line 107).

9. Line 193: please clarify that ‘hypertensive’ refers to measured hypertensive and so there might be some participants with a diagnosis of hypertension who are controlled by medication. This is fine, but it should be clarified.

**Authors’ Response:** We updated the texts (in p.9, lines 199–201) to reflect the reviewer’s comment.

10. Table 3: BMI is not nutritional status, please re-label.

**Authors’ Response:** BMI is a commonly used measure of adult nutritional status for a long time.^[3]^ No changes were made.

11. I would prefer to see SES defined as low, middle, high rather than poor, middle and rich.

**Authors’ Response:** We made the suggested change.

12. The tables require some work. The use of * to indicate p<0.05, <0.001, etc is inappropriate (as already mentioned). There should be tests for trend and it would be better to use floating absolute risks (Easton et al, 1991, Plummer et al 2004) so that appropriate uncertainty is allocated to the reference group and therefore comparisons can be made between any 2 groups and not just with the reference group.

**Authors’ Response:** The revised manuscript does not include the 2011 BDHS data. So, the ‘tests for trend’ is no longer an issue.

In proportional hazards model and conditional logistic regression model for matched case-control studies, floating absolute risk (FAR) is used for estimating the CI for relative risk for each category of a factor variable including the reference group. However, FAR method has several criticisms and we do not find it necessary to achieve the objectives of this study. Therefore, we respectfully disagree with the reviewer and followed the conventional approach (using a reference category) for factor variable.

13. The figures are inadequate. Figure 1 needs N and Cis so that the reader can determine how reliably each prevalence has been determined.

**Authors’ Response:** We updated Figure 1 by adding CI (Fig-1_R1.svg).

14. Figure 2: the y-axis should be ‘% of those with diabetes’. The current tile implies that 100% of the population aged 18 years or older has diabetes.

**Authors’ Response:** We updated Figure 2 after incorporating the reviewer’s comment (Fig-2_R1.svg).

Sincerely,

Karar Zunaid Ahsan (corresponding author)

UNC Center for Health Equity Research,

School of Medicine, the University of North Carolina at Chapel Hill,

323 MacNider Hall 333 South Columbia Street, Chapel Hill, NC 27599-7240, USA.
Email: [zunaid@email.unc.edu](mailto:zunaid@email.unc.edu); Tel: +1 (919) 360-5462

1. Foreit KGF. Addressing Poverty: A Guide for Considering Poverty-Related and Other Inequities in Health. Chapel Hill, NC, 2012.
2. Bellows N, Weinberger M, Reidy M. Using the Demographic Health Survey wealth index to create family planning market segments based on absolute income levels. BMJ Global Health 2020; 5:e002450.
3. Bailey KV, Ferro-Luzzi A. Use of body mass index of adults in assessing individual and community nutritional status. Bulletin of the World Health Organization. 1995;73(5):673.
