# Supplementary material for: Socioeconomic disparities in diabetes prevalence and management among the adult population in Bangladesh
Source: PLoS One. 2022 Dec 20;17(12):e0279228. doi: 10.1371/journal.pone.0279228 (PMC9767371; doi:10.1371/journal.pone.0279228)
Supplement: Attachment — Submitted filename: Response to reviewers_R2.docx [file pone.0279228.s003.docx]

01 September 2022

Mohammad Bellal Hossain, PhD
Academic Editor

PLOS ONE

San Francisco, CA 94111, United States

Subject: Revision of the manuscript PONE-D-21-02392R1

Dear Dr. Hossain:

We would like to submit the revised version (2^nd^ revision) of our research article titled “Socioeconomic disparities in diabetes prevalence and management among the adult population in Bangladesh.” The revised version addresses all the comments by the reviewers. We have made necessary changes as instructed in the paper in track changes. We would also like to take the opportunity to thank the reviewers for their insightful comments.

All the authors have approved this revised version of the manuscript and contributed towards the revision. The authors do not have any conflicts of interest to disclose.

Please find the reviewers’ comments and our responses below:

**Comment from Reviewer 2**

1. My major concern remaining is the multitude of p-values in this paper, with no acknowledgement of or correction for multiple testing.

**Authors’ Response:** For our bivariate analyses (i.e., chi-squared tests), we used the Bonferroni-corrected p-value to examine the association between selected explanatory variables and diabetes prevalence, awareness, treatment, and control (p.13, lines 264–267). Since the multivariable regression analyses in this study were conducted separately for diabetes prevalence, awareness, treatment, and control, we did not correct for multiple testing, and we acknowledged this limitation on p.28, lines 580–583.

**Comments from Reviewer 3**

1. Table 2 does not include information about the sample size used for the test.

**Authors’ Response:** Sample sizes (i.e., the number of respondents) are provided in the last row of Table 2 (p.14).

2. The Chi-square test used for table 2 is not sufficiently explained. Please add more details what has been compared and why are there no alternative tests.

**Authors’ Response:** We thank the reviewer for this constructive comment. We added texts (and references) on pp.9–10, lines 204–218, to explain our choice of chi-squared tests.

3. Table 3 does not include information about the sample size used for the test.

**Authors’ Response:** Sample sizes (i.e., the number of respondents) are provided in the last row of Table 3 (p.17).

4. Add the mathematical definition of aOR (adjusted odds ratio) used in table 3 to the main text. Explain in detail what has been adjusted.

**Authors’ Response:** We added texts explaining aORs on p.15, lines 307–314.

Sincerely,

Karar Zunaid Ahsan (corresponding author)

Assistant Professor,

Public Health Leadership Program,

Gillings School of Global Public Health, University of North Carolina at Chapel Hill,

4111 McGavran-Greenberg Hall, 135 Dauer Drive, Chapel Hill, NC 27599-7469, USA.
Email: [zunaid@email.unc.edu](mailto:zunaid@email.unc.edu); Tel: +1 (919) 360-5462
