# Supplementary material for: Socioeconomic disparities in diabetes prevalence and management among the adult population in Bangladesh
Source: PLoS One. 2022 Dec 20;17(12):e0279228. doi: 10.1371/journal.pone.0279228 (PMC9767371; doi:10.1371/journal.pone.0279228)
Supplement: Attachment — Submitted filename: Response to reviewers_R3.docx [file pone.0279228.s004.docx]

27 November 2022

Mohammad Bellal Hossain, PhD
Academic Editor

PLOS ONE

San Francisco, CA 94111, United States

Subject: Revision of the manuscript PONE-D-21-02392R2

Dear Dr. Hossain:

We would like to submit the revised version (3^rd^ revision) of our research article titled “Socioeconomic disparities in diabetes prevalence and management among the adult population in Bangladesh.” The revised version addresses all the points raised during the review process. We have made all the necessary changes as instructed in the paper in track changes. We would also like to take the opportunity to thank the academic editor and the reviewers for their insightful comments.

All the authors have approved this revised version of the manuscript and contributed towards the revision. The authors do not have any conflicts of interest to disclose.

Please find the reviewers’ comments and our responses below:

**Comment from the Editor**

1. Please note that in your manuscript you have stated (Lines 184 to 187) “Body mass index (BMI) was categorized into three groups—underweight/normal if BMI is less than 25 kg/m2, overweight or obese if BMI is 25 kg/m2 or above, and unknown for the respondents whose height and/or weight data were not available. However, in all of your tables, you have presented it as Nutritional status with the following categories: Underweight, Normal, Overweight/obese, and Unknown. I recommend to use to use BMI; not Nutritional status with the following categories: underweight, normal, overweight or obese, and unknown. It will not be good to merge the underweight with normal.

**Authors’ Response:** We are sorry for this oversight. Based on the reviewer’s comments in an earlier review, we re-analyzed the data using four categories of BMI, and underweight with normal are no longer merged in the current analyses. We have corrected the variable description on p.8, lines 181–182. We also replaced ‘Nutritional status’ with ‘BMI’ throughout the manuscript.

**Comment from Reviewer 2**

1. I still think the authors have not made clear in table 1 that 'Nutritional status' is based on BMI and vice versa in the text that "Body-mass index (BMI) was categorized into three groups..." is referred to as 'Nutritional status' in Table 1.

**Authors’ Response:** We have corrected the variable description on p.8, lines 181–182. We also replaced ‘Nutritional status’ with ‘BMI’ throughout the manuscript.

Sincerely,

Karar Zunaid Ahsan (corresponding author)

Assistant Professor,

Public Health Leadership Program,

Gillings School of Global Public Health, University of North Carolina at Chapel Hill,

4111 McGavran-Greenberg Hall, 135 Dauer Drive, Chapel Hill, NC 27599-7469, USA.
Email: [zunaid@email.unc.edu](mailto:zunaid@email.unc.edu); Tel: +1 (919) 360-5462
